# Dentinogenic Ghost Cell Tumor in a Sumatran Rhinoceros

**DOI:** 10.3390/ani11041173

**Published:** 2021-04-20

**Authors:** Annas Salleh, Zainal Z. Zainuddin, Reza M. M. Tarmizi, Chee K. Yap, Chian-Ren Jeng, Mohd Zamri-Saad

**Affiliations:** 1 Department of Veterinary Laboratory Diagnosis, Faculty of Veterinary Medicine, Universiti Putra Malaysia, Serdang 43400, Selangor, Malaysia; mzamri@upm.edu.my; 2 Borneo Rhino Alliance, c/o Faculty of Sciences and Natural Resources, Universiti Malaysia Sabah, Kota Kinabalu 88400, Sabah, Malaysia; zainalz.bora@gmail.com (Z.Z.Z.); reza2727@gmail.com (R.M.M.T.); kcyap@upm.edu.my (C.K.Y.); 3 Graduate Institute of Molecular and Comparative Pathobiology, School of Veterinary Medicine, National Taiwan University, Taipei 106216, Taiwan; crjeng@ntu.edu.tw

**Keywords:** dentinogenic ghost cell tumor, odontogenic ghost cell lesion, *Sumatran rhinoceros*, *Dicerorhinus sumatrensis*, immunohistochemistry, special stain

## Abstract

**Simple Summary:**

A dentinogenic ghost cell tumor is an odontogenic ghost cell lesion of the maxilla and mandible. It is a rare tumor that has been described in humans. This work describes the clinical and pathological findings of an advanced stage of a dentinogenic ghost cell tumor, a type that has not previously been described in veterinary medicine. The advanced stage of this tumor led to the observation of aberrant keratinization, characterized by ghost cells and numerous islands of dentinoid formation. Diagnosis was made with the aid of routine histology, special histochemistry, immunohistochemistry, and classification and features from human oncology as a reference.

**Abstract:**

An adult female Sumatran rhinoceros was observed with a swelling in the left infraorbital region in March 2017. The swelling rapidly grew into a mass. A radiograph revealed a cystic radiolucent area in the left maxilla. In June 2017, the rhinoceros was euthanized. At necropsy, the infraorbital mass measured 21 cm × 30 cm. Samples of the infraorbital mass, left parotid gland, and left masseter muscle were collected for histopathology (Hematoxylin & Eosin, Von Kossa, Masson’s trichrome, cytokeratin AE1/AE3, EMA, p53, and S-100). Numerous neoplastic epithelial cells showing pleomorphism and infiltration were observed. Islands of dentinoid material containing ghost cells and keratin pearls were observed with the aid of the two special histochemistry stains. Mitotic figures were rarely observed. All the neoplastic odontogenic cells and keratin pearls showed an intense positive stain for cytokeratin AE1/AE3, while some keratin pearls showed mild positive stains for S-100. All samples were negative for p53 and S-100 immunodetection. The mass was diagnosed as a dentinogenic ghost cell tumor.

## 1. Introduction

In humans, a few types of tumors are identified as odontogenic ghost cell lesions (OGCL) of the maxilla and mandible. This includes calcifying odontogenic cysts (COC), dentinogenic ghost cell tumors (DGCT), and ghost cell odontogenic carcinoma (GCOC) [1]. DGCT is a benign but locally infiltrative neoplasm of odontogenic epithelium. It is a rare tumor in humans with very limited reports. A ghost cell is an enlarged epithelial cell having an eosinophilic cytoplasm with a faint nucleus outline or no nucleus [2]. It is associated with a marked aberrant keratinization. DGCT has been described as a rare form of ghost cell lesion, accounting for 3–5% of all cases involving ghost cell lesions [3,4].

For OGCL in humans, the prognosis and recurrence rate may differ according to the type of tumor. For COC, prognosis is considered excellent and the recurrence rate is low. When recurrence of COC occurs, it typically involves elderly persons [5]. Recurrence in young persons is rarely reported [6]. For DGCT, reports on recurrence rates range between 33% and 73% [7]. Surgical removal of DGCT involving an extensive procedure usually results in a low recurrence rate, while simple enucleation of the tumor usually results in a higher recurrence rate. Recurrence may occur within 5 to 10 years [8]. GCOC has a 73% five-year survival rate, and recurrence is reported to be common [9]. 

To our knowledge, DGCT has never been documented in animals. This article reports the first case of DGCT in an animal.

## 2. Description of the Case

A female Sumatran rhinoceros (*Dicerorrhinus sumatrensis*) weighing 508 kg and estimated to be between 25 and 30 years old was managed in a one-hectare forested paddock at the Tabin Wildlife Reserve, Sabah, Malaysia. In January 2017, it showed signs of difficulty in mastication, especially chewing on larger stems. Subsequently, in February 2017, it developed a 5 cm left unilateral, infraorbital and maxillary swelling with epiphora. It was treated with oral flunixin meglumine (Banamine^®^ at 1500 mg per day for 3 days, and oral amoxicillin and clavulanate potassium (Augmentin^TM^) for five consecutive days. However, within a month, the swelling rapidly developed into a firm mass measuring about 15 cm in diameter, which later ruptured to discharge a mucopurulent exudate. In addition to wound cleaning twice a day, the rhinoceros was treated with oral amoxicillin and clavulanate potassium (Augmentin^TM^) at 25 mg/kg for 5 days, and parenteral dexamethasone (Dexadreson^®^) at 0.1 mg/kg intramuscularly for 3 days. Despite the treatment, the wound did not show any improvement and eventually became a 5 cm over-granulated open wound with blood-tinged nasal discharge from the left nostril. At this point, the appetite and body weight were slightly reduced, while the right jaw was predominantly used for mastication.

**Figure 1:**
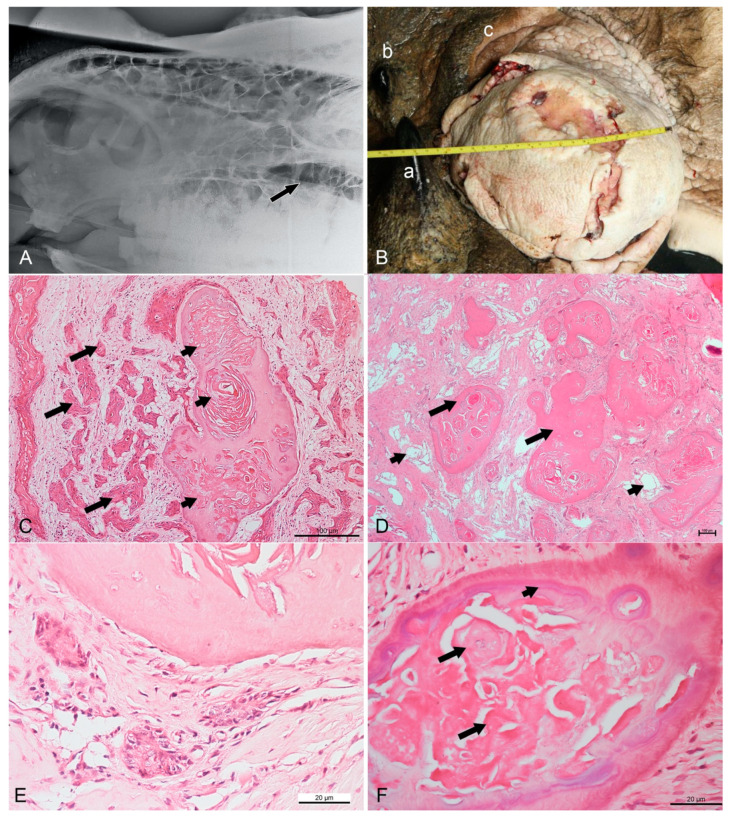
Radiographic, gross, and routine histopathological findings in a Sumatran rhinoceros with a dentinogenic ghost cell tumor. (**A**) Left-lateral view radiograph taken in April 2017 showing a unilocular radiolucent cyst (arrow) at the left maxilla. (**B**) The infraorbital mass in June 2017 measuring 21 cm × 30 cm. a: anterior horn, b: posterior horn, c: left upper eyelid. (**C**) Nests of neoplastic squamous cells (long arrows) surrounded by substantial compact fibrous stroma. Note the formation of keratin pearls (short arrows) embedded in a dentinoid material. HE (hematoxylin and eosin), bar = 100 µm. (**D**) Numerous islands of dentinoid material (long arrows) surrounded by loose and vascularized stroma (short arrows). HE, bar = 100 µm. (**E**) Neoplastic cells showing pleomorphism with basaloid- or stellate-reticulum-like appearance arranged in a nest. Vesicular nuclei can be observed. HE, bar = 20 µm (**F**) Ghost cell (long arrows) at the center of dentinoid material with cementum-like appearance (short arrow). HE, bar = 20 µm.

*Staphylococcus* sp. was isolated from the swab sample of the open wound, while an antibiotic sensitivity test showed resistance to amoxicillin-clavulanate acid but susceptibility to enrofloxacin and cephalosporin. In April 2017, a radiograph revealed a unilocular radiolucent area surrounding the 2nd and 3rd maxillary cheek teeth, suggestive of a cyst (Figure 1A). This cyst was connected to the paranasal sinuses by an oronasal fistula. A radiopaque fragment was noted dorsal to the 3rd maxillary cheek tooth, indicating a fracture of alveolar bone. The rhinoceros was orally treated with dexamethasone, Augmentin^TM^, lactated Ringer’s solution, dextrose, Duphalyte, vitamin K, iron supplement, and phenylbutazone.

Dental extraction surgery was performed with peri-operative treatment comprising flunixin meglumine and enrofloxacin. Three cheek teeth (1st, 2nd, and 3rd cheek teeth) were successfully extracted in the surgery. All the extracted teeth had yellowish expansile solid masses around the roots. However, the oronasal fistula was not examined, as it could not be reached through the alveolar opening. For post-operative treatment, phenylbutazone, enrofloxacin, ceftiofur, and oral rinse were administered.

Thirty minutes after the recovery from anesthesia, the animal regained normal appetite. Wound cleaning, mouth wash, and parenteral enrofloxacin once daily, every other day were continued. However, the open wound, nasal discharge, and epiphora persisted. Thus, the antibiotic was changed to ceftiofur on day 8 after dental extraction. The bodyweight increased to 512 kg 7 days after the surgery. The intraoral granulation tissue eventually subsided. However, between May and June 2017, the animal showed occasional epistaxis and dyspnea, while the cutaneous mass aggressively grew larger. The rhinoceros was euthanized by intravenous administration of detomidine, ketamine, and pentobarbitone.

During the post-mortem examination, a tissue mass was visible around the dental extraction site, with the remaining 2nd and 3rd molars having enormous amounts of the expansile solid mass around the crowns and roots. The skin around the open wound was edematous and swollen. The infraorbital mass measured 21 × 30 cm (Figure 1B), with several open wounds of 1 to 7 cm in diameter. The mass extended ventrally and dorsal into the eyes. The size and color of the left masseter muscles were darker compared to the opposite side, suggestive of degenerative changes. Fistula between the maxilla and infraorbital mass was noted, while the left parotid gland was gritty with whitish spots. No metastasis to either adjacent or distant organs was observed. Samples from the infraorbital mass, left parotid gland, and left masseter muscle were collected and fixed in 10% neutral-buffered formalin, routinely processed, and stained with hematoxylin and eosin (HE), special histochemical Masson’s trichrome and Von Kossa stains, and immunohistochemistry was conducted for detection of cytokeratin AE1/AE3, epithelial membrane antigen (EMA), p53, and S-100.

**Figure 2:**
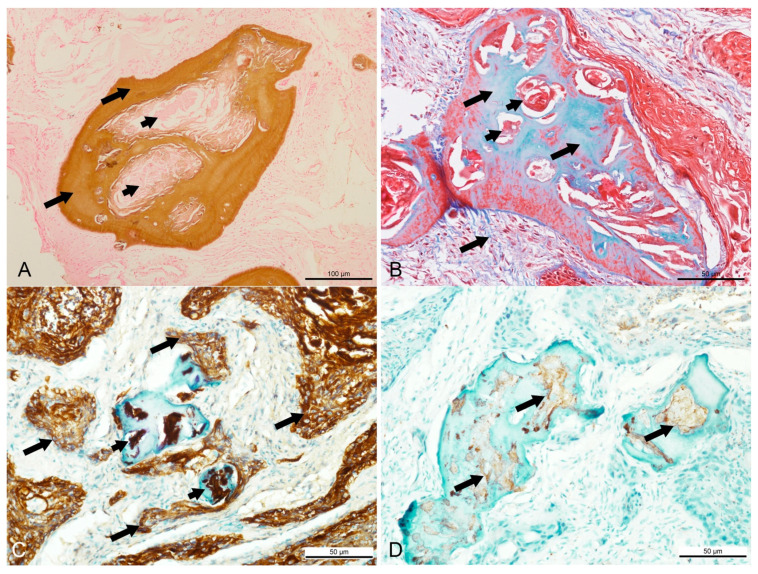
Special histochemical and immunohistochemistry findings in a Sumatran rhinoceros with a dentinogenic ghost cell tumor. (**A**) Brown stain of Von Kossa indicating the presence of calcium in the dentinoid material (long arrow), while most keratin pearls were devoid of calcium (short arrows). Von Kossa, bar = 100 µm. (**B**) Bone tissue stained in blue and keratin stained in red with Masson’s trichrome. Masson’s trichrome, bar = 50 µm. (**C**) Intense intracytoplasmic staining for cytokeratin AE1/AE3 in the neoplastic squamous cells (long arrows) and keratin pearls (short arrows). AE1/AE3, bar = 50 µm. (**D**) Mild positive staining for S-100 in the ghost cells and keratin (arrows) located inside the dentinoid material. S-100, bar = 50 µm.

In the infraorbital mass, islands of neoplastic epithelium of various sizes were observed embedded or infiltrated in substantial amounts of either compact or loose fibrous stroma (Figure 1C). In some areas, the stroma was extensively loose with increased vascularization (Figure 1D). The neoplastic cells showed an infiltrative growth pattern arranged in strands, unsuccessful anastomosing, or medusa-like patterns. A long trabecular arrangement of tumor cells was observed. Multifocal squamous metaplasia or keratin-like material deposition was noted in the centers of the tumor islands. The tumor cells could be seen surrounding and embedded in numerous islands of dentinoid material. In addition, accumulation of pale eosinophilic ghost cells and a whirl-like arrangement of keratin-like material infiltrating the dentinoid material were noticeable. At high magnification, the neoplastic cells showed pleomorphism with a basaloid- or stellate-reticulum-like appearance with vesicular nuclei, usually arranged in a nest (Figure 1E). Ghost cells, characterized by large, eosinophilic cells that contained either the outline of a nucleus or no nucleus, were present at the cementum-like appearance of the dentinoid materials (Figure 1F). Mitoses were occasionally seen. The mitotic count, determined using a previously described method, was a low count of 2 [10]. The dentinoid was further confirmed by positive staining using Von Kossa stain to indicate the presence of calcium, and blue staining by Masson’s trichrome stain. Most of the keratin and ghost cells lacked calcium, as indicated by the negative staining by Von Kossa stain (Figure 2A) and red staining by Masson’s trichrome stain (Figure 2B). Some keratin pearls were observed without dentinoid formation, but they were surrounded by substantial amounts of neoplastic epithelial cells. The left masseter muscle was mildly degenerated but showed no evidence of invasion by neoplastic cells, while the left parotid gland was severely calcified.

The neoplastic epithelial cells showed intense intracytoplasmic immunodetection of cytokeratin AE1/AE3 but were negative for EMA and S-100. All keratin pearls, including those found inside the dentinoid material, and most of the ghost cells, showed intense staining with cytokeratin AE1/AE3 (Figure 2C), mild staining against S-100 (Figure 2D), and negative against p53 and EMA.

The differential diagnoses for this case included ghost cell odontogenic carcinoma (GCOC), dentinogenic ghost cell tumor (DGCT), craniopharyngioma, primary intraosseous squamous cell carcinoma (PIOSCC), squamous cell carcinoma (SCC), and ameloblastoma. The radiology and histopathology examinations established the diagnosis of DGCT.

## 3. Discussion

OGCL are considered challenging to diagnose, as COC, DGCT, and GCOC have similar histological features [11]. Diagnosis of DGCT in this rhinoceros was largely made based on the histological and immunohistochemical features from human oncology and pathology as compiled in Table 1. From the differential diagnoses, PIOSCC, SCC and ameloblastoma were ruled out, as these tumors do not feature ghost cell lesions [12]. GCOC was ruled out mainly by the fact that histopathological examination showed low mitotic activity, suggestive of a benign cellular status. Furthermore, it did not invade adjacent tissues, lacked necrosis, had pleomorphic neoplastic cells, and the immunohistochemistry for p53 was negative. Although about 30% of GCOC may show negativity for p53, it has been reported that the diagnosis of GCOC versus DGCT should be largely based on p53 positivity [3,13]. Formation of dentinoid material in craniopharyngioma is extremely rare. If present, these dentinoid materials are described as not obvious [14], so craniopharyngioma was ruled out. From this case and bibliographical review, it was stated that differentiating DGCT, GCOC, and other differential diagnoses based on the epithelial histological and immunochemical features can be difficult. The reason for this is that they may show similar epithelial features ranging from palisading columnar (resembling ameloblastoma) to basaloid (resembling squamous epithelium) formations. The presence of foreign body giant cells has been reported in both DGCT and GCOC [15,16]. Because of the rarity of OGCL and the many synonyms for each OGCL neoplasm, available data pertaining to their immunohistochemical characteristics may be difficult to access.

In general, DGCT more commonly occurs in the posterior maxilla and mandible. A slight predilection for the mandible has been reported, where 53% of DGCT occurs in the mandible [3]. Two variants of DGCT, namely, central and peripheral, have been described [26]. Central DGCT, the more common of the two, is a locally invasive intraosseous tumor, whereas peripheral DGCT is a non-invasive extraosseous tumor [27]. In most cases of central DGCT, the radiographic features are unilocular with a mixture of radiolucent and radiopaque or only radiolucent lesions [3]. It is unfortunate that no sample from the maxillary cyst was collected and examined in this case. The radiographic observation of mandibular cyst in this rhinoceros suggested that this case involved a central DGCT.

Cases of other OGCL in animals have been previously reported, such as epithelial ghost cells and dentinoid material in rats with odontogenic tumors [28]. Another report involved a Bengal tiger (*Panthera tigris tigris*), wherein only a few ghost cells and some keratin were observed in a mandibular mass. However, no formation of dentinoid material was observed. That case was diagnosed as calcifying epithelial odontogenic tumor [29]. It is possible that the lack of reports of GCOC is due to the rarity of the tumor or the general lack of classification of odontogenic tumors in veterinary medicine [30]. Histological similarities were observed with the previously reported odontogenic tumors in rats, wherein no ameloblastoma-like tumor cells were seen and ovoid neoplastic epithelial cells predominated [28]. But this was very different from DGCT in humans, wherein ameloblastomatous proliferation is typically obvious [3,17].

In megavertebrates, oral and facial proliferative lesions have been previously reported. This includes cases of gingivitis, tooth root abscessation, and SCC [31,32,33]. It is important to conduct routine clinical examinations and detailed histopathological examinations to properly diagnose these lesions. Despite its rarity, OGCL should be considered in cases of oral and facial proliferative lesions in megavertebrates and animals in general.

## 4. Conclusions

This is the first report of DGCT in veterinary medicine. The diagnosis of DGCT in this case was made based on routine histopathology, special histochemistry, and immunohistochemistry with human oncology and pathology as a reference. The histopathology and immunohistochemistry of DGCT in this rhinoceros match the majority of descriptions of DGCT in humans.

## Figures and Tables

**Table 1:** Summary of histological and immunohistochemical features of COC, DGCT, and GCOC in humans.

	COC	DGCT	GCOC	References
Histological Features				
Cyst component	Main	Occasional	Occasional	[17]
Epithelium	Mainly cystic Palisading columnar cells resembling ameloblastoma	Tumorous and occasionally cystic Ameloblastous or basaloid	Tumorous and rarely cystic Uniform small basaloid, with round or vesicular nuclei	[3,17]
Ghost Cell	Consistent	Marked	Predominant	[17]
Calcification	Frequent	Occasional	Rare	[17]
Dentinoid Material	None	Predominant	Rudimentary	[17]
Cellular status	Benign	Benign	Malignant	[17]
Mitosis	Present	Rare	Frequent	[3]
Recurrence	Rare	Rare	Frequent	[17]
Immunohistochemistry Features				
Cytokeratin AE1/AE3	+	+	+	[18,19]
Beta catenin	+	+	+	[20,21]
S-100	+/−	+	+/−	[18,22,23,24]
EMA	n/a	n/a	−	[18]
p53	n/a	+/−	+/− (>70% of cases show +)	[3,18,19,25]

## Data Availability

Not applicable.
